# Monomer-dependent secondary nucleation in amyloid formation

**DOI:** 10.1007/s12551-017-0289-z

**Published:** 2017-08-15

**Authors:** Sara Linse

**Affiliations:** Chemical Centre, Biochemistry & Structural Biology, Lund University, P O Box 124, S22100 Lund, Sweden

**Keywords:** Monomers, Secondary nucleation, Amyloid formation, Inhibitors, Therapeutic developments

## Abstract

Secondary nucleation of monomers on the surface of an already existing aggregate that is formed from the same kind of monomers may lead to autocatalytic amplification of a self-assembly process. Such monomer-dependent secondary nucleation occurs during the crystallization of small molecules or proteins and self-assembled materials, as well as in protein self-assembly into fibrous structures. Indications of secondary nucleation may come from analyses of kinetic experiments starting from pure monomers or monomers supplemented with a low concentration of pre-formed aggregates (seeds). More firm evidence requires additional experiments, for example those employing isotope labels to distinguish new aggregates arising from the monomer from those resulting from fragmentation of the seed. In cases of amyloid formation, secondary nucleation leads to the formation of toxic oligomers, and inhibitors of secondary nucleation may serve as starting points for therapeutic developments. Secondary nucleation displays a high degree of structural specificity and may be enhanced by mutations or screening of electrostatic repulsion.

## Introduction

**Fig. 1:**
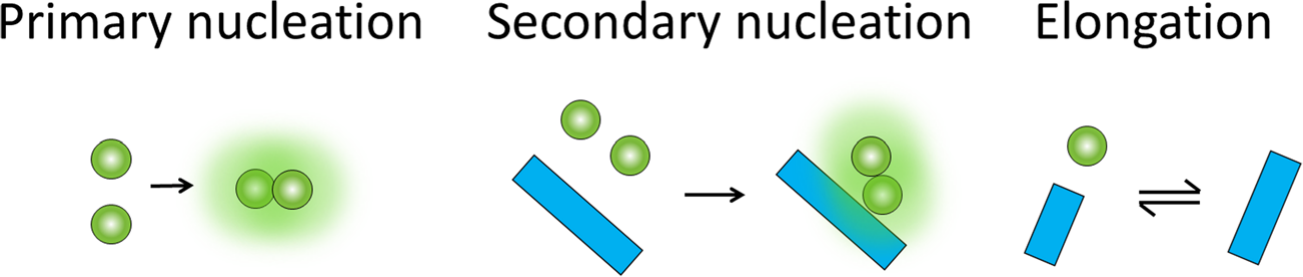
Minimal set of microscopic steps that can globally fit all macroscopic kinetic profiles for Aβ42 fibril formation as a function of time and initial monomer concentration for non-seeded as well as seeded samples. Primary nucleation (*left*) involves monomer only, and the nucleus is defined as a species that grows faster by monomer addition compared to its dissociation back to monomer. Secondary nucleation (*middle*) produces nuclei from monomers in a reaction catalysed by already existing aggregates of the same kind of monomer. Elongation (*right*) leads to the growth of fibrillar aggregates by monomer addition

Monomer-dependent secondary nucleation is defined as a process whereby monomers form a nucleus on the surface of an already existing aggregate formed from the same kind of monomers (Fig. 1). Primary nucleation, in contrast, involves only monomers but may occur in bulk solution (homogeneous nucleation) or at a surface (heterogeneous nucleation) of some other substance or even at the air–water interface. Secondary nucleation is thus distinct from heterogeneous primary nucleation.

Secondary nucleation leads to propagation of three-dimensional crystals of proteins and small molecules (Botsaris 1976; Kondepudi et al. 1990; Lindenmeyer 1977; Miers and Isaac 1906). Monte Carlo simulations have revealed secondary nucleation in crystallization of the simplest possible model system—that of Lennard Jones particles—and shown that the level of super-saturation of the system controls whether the reaction is dominated by primary nucleation, elongation or surface-catalysed secondary nucleation (Anwar et al. 2015). Secondary nucleation may be utilized as an aid in crystallization procedures (Mullin 2001; Cubillas and Anderson 2010). An old crystal, a seed, is introduced into a drop of supersaturated monomer solution. At the intermediate level of supersaturation, the monomers may nucleate on the surface of the seed and thereby form new crystals having the same morphology as the seed. The newly nucleated crystals grow by monomer addition, thereby amplifying the initial “hit” into multiple crystals with identical packing. In some applications, for example production of synthetic diamonds, the conditions are instead chosen to suppress secondary nucleation to favor growth of a small number of large crystals rather than a large number of small ones (Jiang and Tzeng 2011).

Secondary nucleation may also lead to the propagation of elongated or fibrous aggregates, which may be viewed as two-dimensional or even one-dimensional crystals. In 1985, Ferrone et al. (1985) reported that aggregates of sickle-cell haemoglobin catalyse on their surface the nucleation of new aggregates from protein monomers. Monomer-dependent secondary nucleation in amyloid formation has been inferred for several proteins, including insulin (Foderà et al. 2008) and islet amyloid poly peptide (IAPP; Ruschak and Miranker 2007), both involved in diabetes, amyloid-β peptide (Aβ; Cohen et al. 2013) involved in Alzheimer’s disease and α-synuclein (α-syn; Buell et al. 2014, Gaspar et al. 2017) involved in Parkinson’s disease.

**Fig. 2:**
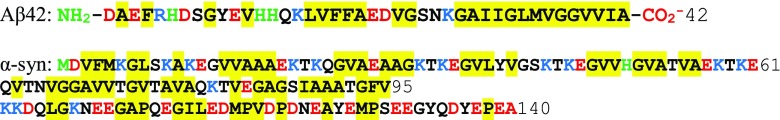
The amphiphatic amino acid sequences of the 42-residue amyloid β peptide (*Aβ42*) and α-synuclein (*α-syn*). The sequences are shown with the following colour codes: *red* negatively charged, *blue* positively charged, *green* titrating around neutral pH, *yellow* hydrophobic residue

In this article we highlight some of the recent findings on monomer-dependent secondary nucleation of Aβ and α-syn. Aβ is proteolysed from the amyloid precursor protein by the β- and γ-secretases (Glenner and Wong 1984). The peptide is present at nanomole or sub-nanomole concentrations in several body fluids and may also enter cells. According to the amyloid cascade hypothesis, amyloid formation of Aβ, and subsequently of protein tau, contributes to the pathology of Alzheimer’s disease (Beyreuther and Masters 1991; Hardy and Allsop 1991; Selkoe 1991; Hardy and Higgins 1992). α-Syn (Maroteaux et al. 1988; Tuttle et al. 2016) is an intrinsically unfolded protein of 140 residues, present at approximately 20 μM concentration in multiple cell types. The so-called Lewy bodies, plaques of the protein α-syn, are hallmarks of Parkinson’s disease. α-Syn has considerable affinity for negatively charged phospholipid membranes (Davidson et al. 1998), which may promote heterogeneous primary nucleation (Galvagnion et al. 2015). It is also thought to be involved in synaptic functions (Clayton and George 1999). The amphiphatic amino acid sequences of Aβ42 and α-syn are shown in Fig. 2, with colour coding indicating the charge of the various sequences.

## Amyloid aggregates

While the folded structure and function of the native state of a protein is dictated by its amino acid sequence (Anfinsen 1973), proteins can adopt an alternative structure that is highly similar irrespective of sequence—the amyloid fibril (Chiti and Dobson 2006, 2017; Eisenberg and Jucker 2012; Knowles et al. 2014). This may indeed be a generic structure that can be formed by any sequence, at least under some solution conditions (Dobson 2001). Amyloid fibrils have a highly repetitive packing of multiple identical protein chains in the extended β-sheet (Astbury et al. 1935) and are aggregates of protein alone or protein + lipids (Gellermann et al. 2005). High-resolution structures of a range of amyloid fibrils have been solved using X-ray diffraction, X-ray crystallography, solid-state nuclear magnetic resonance spectroscopy and cryo-electron microscopy, including those for Aβ (Antzutkin et al. 2000; Schütz et al. 2015; Colvin et al. 2016; Wälti et al. 2016; Qiang et al. 2017), tau (Fitzpatrick et al. 2017), α-syn (Rodriguez et al. 2015,) and IAPP (Wiltzius et al. 2008). Pre-fibrillar aggregates include several oligomeric forms. The word oligomer may have many definitions. A common definition of oligomer is an aggregated species of two or more monomers which grows at lower rate than a fibril. The structure of such oligomers is less ordered than that of mature fibrils, and due their low concentration compared to that of monomers and fibrils, they may require smart trapping strategies to study their structure (Lendel et al. 2014). When using separation methods such as gel filtration (see following sections), it may be more convenient to use an operational definition of oligomers to include species of a certain size range, such as those that elute between the void and the monomer peak.

## Discovery of monomer-dependent secondary nucleation of the amyloid β peptide Aβ42

**Fig. 3:**
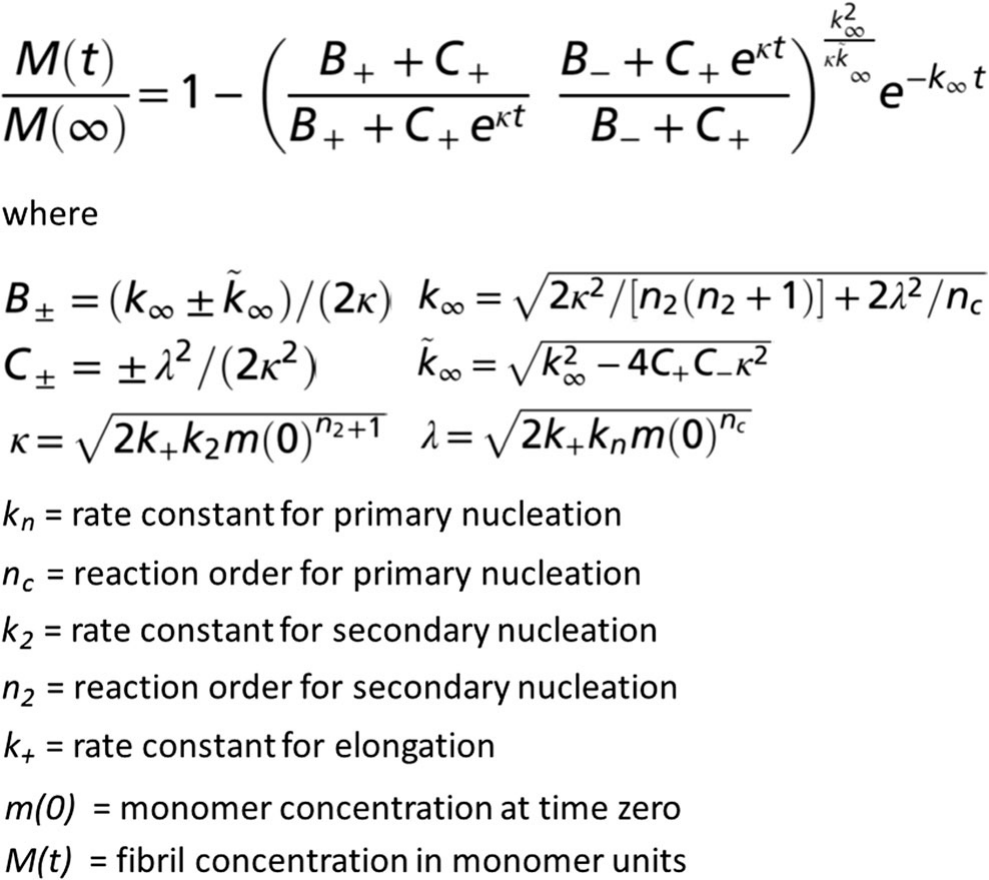
Master equation used to analyse aggregation data. The master equation shown is valid for reactions governed by primary nucleation, elongation and secondary nucleation (Cohen et al. 2013). The master equation for reactions governed by primary nucleation and elongation is obtained by setting k_2_ = 0. The master equation for reaction governed by primary nucleation, elongation and fragmentation can be obtained by setting *n*_2_ = 0 and replacing k_2_ with k_−_, the rate constant for fragmentation

**Fig. 4:**
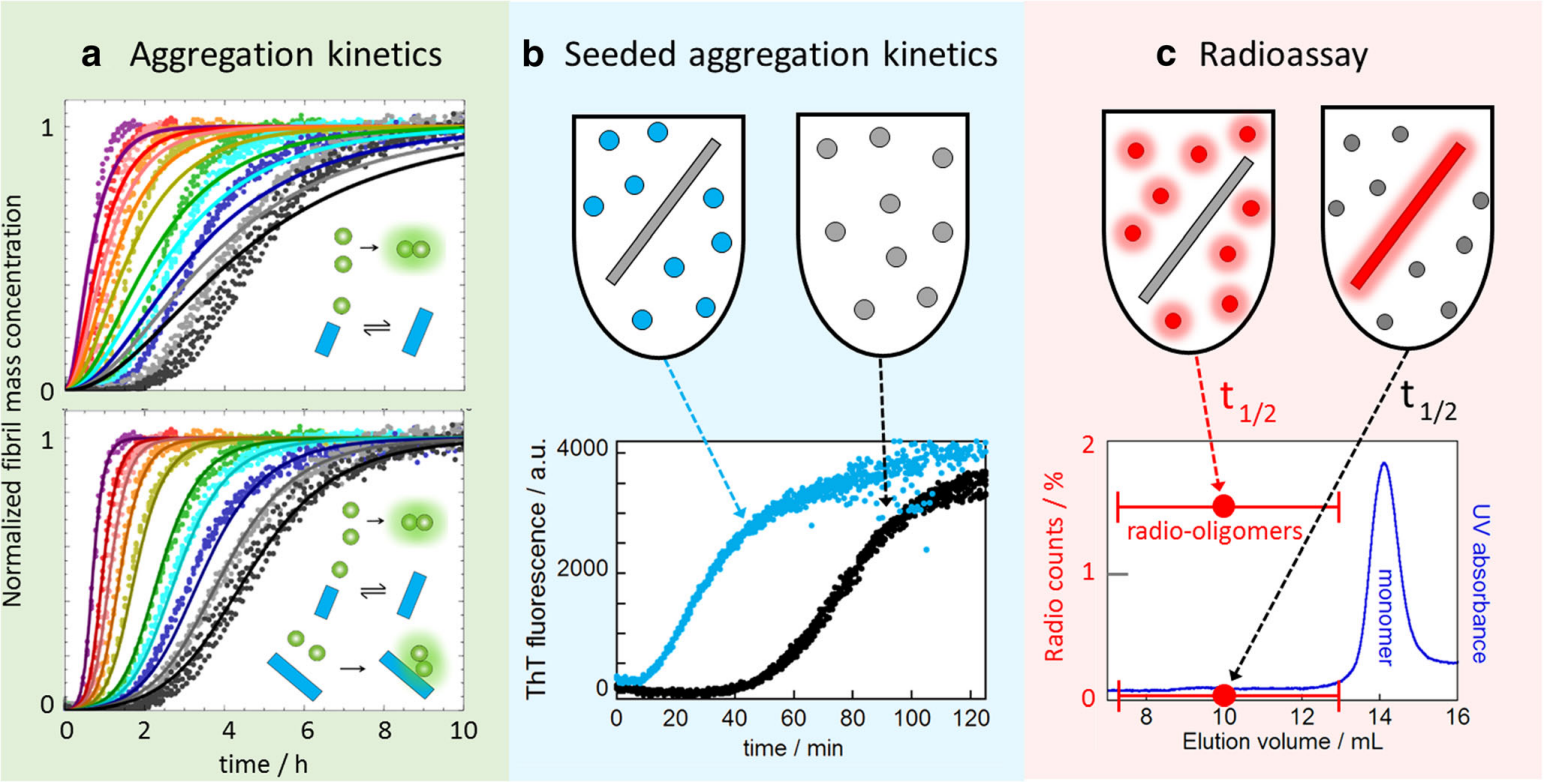
Discovery of monomer-dependent secondary nucleation. All data were acquired in a solution of 20 mM sodium phosphate, 0.2 mM EDTA, 0.02% NaN3, pH 8.0, at 37 °C under quiescent conditions using thioflavin T fluorescence as a reporter of fibril formation. **a** Fibril formation kinetics for Aβ42 starting from solutions of freshly isolated monomer. Each *colour* represents a separate monomer concentration, and quadruplicate experiments are shown. *Top panel* shows the best possible fit for a model with primary nucleation and elongation. *Bottom panel* shows the improved fit when also secondary nucleation of monomers on aggregate surface is included. **b** Shortening of the lag phase in the presence of seeds signifies a secondary process. Fibril formation is monitored for solutions that initially contain 3 μM Aβ42 monomer and no (*black*) or 30 nM pre-formed seeds (*blue*) (seed concentration in is monomer equivalents). **c** Radio-assay using Aβ42 produced with [^35^S]methionine shows that oligomers are generated from monomer in a reaction catalysed by the seed fibrils. One sample (*left*) contained 5 μM [^35^S]Aβ42 monomer and 50 nM unlabeled seed. The other sample (*right*) contained 5 μM unlabelled Aβ42 monomer and 50 nM ^35^S-labelled seed. Both samples were incubated at 37 °C until reaching the half-time (*t*_*1/2*_), i.e. the point in time where half the monomers had converted to fibrils. Fibrils were removed by centrifugation and the supernatant subjected to gel filtration on a Superdex 75 column. Oligomer fractions, which eluted before the monomer, were collected, lyophilized, dissolved and mixed with scintillation liquid for radio-decay counting, in comparison with a dilution series of the initial monomer solution. No radio-oligomers were detected for the sample with unlabelled Aβ42 monomer and ^35^S-labelled seed, whereas the sample with the [^35^S]Aβ42 monomer and unlabeled seed generated a high concentration of radio-oligomers corresponding to approximately 1.5% of the total monomer concentration

Monomer-dependent secondary nucleation of the 42-residue amyloid β peptide (Aβ42) was discovered as late as during the current decade. The first indication of the existence of such a process came from the discovery that using master equations solved for the coupled differential equations describing primary nucleation and elongation, or primary nucleation, elongation and fragmentation (Knowles et al. 2009) (Fig. 3) failed to reproduce experimental data on the concentration–dependent time course of amyloid fibril formation (Fig. 2a, top; Cohen et al. 2013). While the data could be reproduced by a model that also includes nucleation of monomers on the surface of fibrils formation (Fig. 4a, bottom) (Cohen et al. 2013; see also Cohen et al. 2011a, b, c, 2012), this is only an indication of the existence of secondary nucleation, which was tested by predicting the outcome of new experiments that were then conducted. One prediction was that the addition of small amounts of preformed fibrils (seeds), of a quantity so small that the sigmoidal-like shape of the growth curve is conserved, would cause a shortening of the lag phase in a manner dependent on the seed concentration. This was indeed found (Fig. 4b) (Cohen et al. 2013). Finally, radio-isotope labelling was used to pinpoint the origin of new small aggregates in the seeded reaction. Radioactive oligomers (3–20 mers) were only found when radio-active monomer was mixed with unlabelled seeds, but not when unlabelled monomer was mixed with radioactive seeds (Fig. 4c), demonstrating that new aggregates are generated from monomer in a seed-catalysed reaction, rather than being breakdown products due to fragmentation of the seeds. Thus, while kinetic analysis indicates that the aggregation mechanism is dominated by a secondary pathway, seeded experiments and the use of specific isotope labelling identifies the secondary pathway as a process that produces new aggregates from monomer on the surface of fibrils (Cohen et al. 2013). Seeding experiments have also been used to quantify fibril concentration as a function of time during the lag phase of an amyloid formation reaction of Aβ42 (Arosio et al. 2014).

## Secondary nucleation generates the majority of toxic oligomers

According to the current consensus, the amyloid fibrils are not toxic per se, but fibrils might still be disease-relevant species (Tipping et al. 2015). Many studies have indicated that toxicity is mainly associated with protein oligomers (Bucciantini et al. 2002; Walsh et al. 2002; Haass and Selkoe 2007). Most intriguingly, toxicity seems to arise during the aggregation process in a reaction involving both fibrils and monomers (Jan et al. 2011, Cohen et al. 2013, 2015). Electrophysiology measurements in the rat brain slices of γ oscillations, a process involved in memory and learning, revealed a strong toxic effect from species produced due to secondary nucleation of monomers on fibril surfaces (Cohen et al. 2015).

**Fig. 5:**
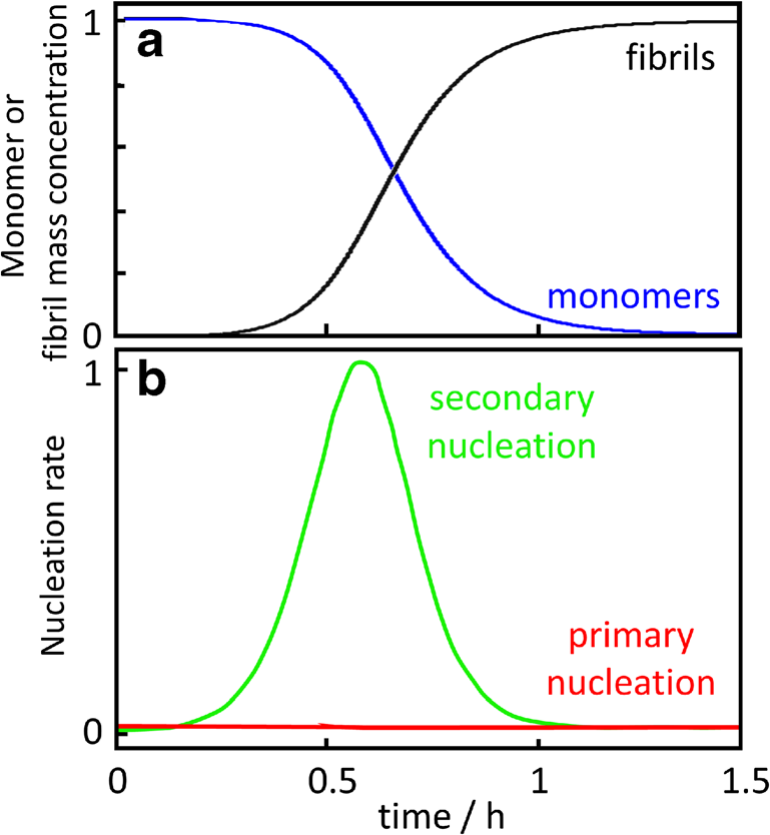
Nucleation rate. **a** Monomer (*blue*) and fibril (*black*) concentration as a function of time for a reaction stating from 4 μM Aβ42 in 20 mM sodium phosphate, 0.2 mM EDTA, 0.02% NaN3, pH 8.0 at 37 °C under quiescent conditions. The curves were calculated using the rate constants determined by Cohen et al. (2013): k_n_ = 3^.^10^−1^ M^−1^ s^−1^, k_2_ = 1^.^10^4^ M^−1^ s^−1^, k_+_ = 3^.^10^6^ M^−1^ s^−1^. **b** Rates of primary (*red*) and secondary (*green*) nucleation calculated as a function of time using the rate constants and concentrations from **a** as: primary nucleation rate = k_n_ [monomer]^2^; secondary nucleation rate = k_2_ [fibril] [monomer]^2^

Using rate constants measured for primary nucleation, secondary nucleation and elongation, one may calculate the nucleation rate as a function of time for solutions, which at time zero contain monomer only at a defined concentration (Fig. 5). Primary nucleation dominates at time zero when only the monomer is present, but secondary nucleation takes over and dominates the process soon after the first aggregates have formed (Arosio et al. 2015). Indeed, secondary nucleation generates many orders of magnitude more oligomers than primary nucleation over the time course of the reaction. The rate of primary nucleation, which depends on monomer concentration only, reaches its maximum at time zero (Fig. 5b, red line) and remains relatively constant over the entire lag phase where the monomer concentration remains almost intact (Fig. 5a, blue line). The rate of secondary nucleation (Fig. 5b, green line) depends on both aggregate and monomer concentration and has reaches its maximum close to the mid-point of the growth phase of the macroscopic aggregation curve, where both monomer and fibril concentration are close to 50% of the total monomer concentration (Fig. 5a; crossover of blue and black line).

## Intra-cellular targets of toxic oligomers during an aggregation reaction

**Fig. 6:**
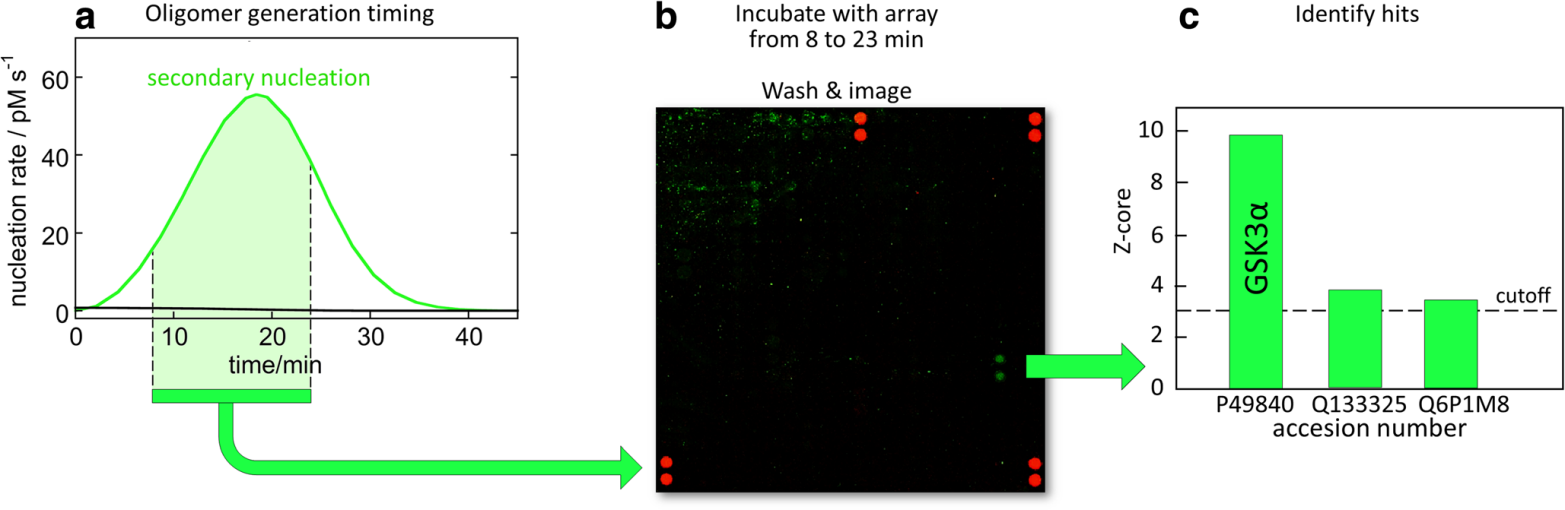
Discovery of intracellular targets for Ab42 oligomers. **a** The rate constants determined in physiological salt buffer at 37 °C (Meisl et al. 2017) were used to calculate the rates of primary (*black*, close to *baseline*) and secondary (*green*) nucleation as a function of time starting from 5 μM Aβ42. It was anticipated that most of the toxic oligomers are generated between 8 and 23 min into the reaction zero. Alexa488–Aβ42 and Aβ42 were separately isolated as monomers by gel filtration, mixed 1:5 and then incubated for 8 min at 37 °C, after which a protein array microscope slide with 9600 human proteins in duplicate (Protoarray® microarray; Invitrogen, Carlsbad, CA) was placed on top of the reaction solution, followed by another 15 min of incubation, then washing and imaging of the array. *Green spots* Bound Aβ42, *red spots* pre-printed guiding spots. Only one duplicate spot was found with a signal significantly above the noise, corresponding to glycogen synthase kinase 3α (Dunning et al. 2016)

Dunning et al. (2016) performed protein array screening of intracellular targets of Aβ42 oligomers generated by secondary nucleation during an on-going aggregation reaction with the aim to cover the time-frame during which the majority of toxic oligomers are generated. Using the rate constants, determined in physiological salt buffer at 37 °C, these authors calculated that most of the toxic oligomers would be generated between 8 and 23 min of a reaction starting from 5 μM monomer at time zero (Fig. 6a). An upside–down orientation, with the array placed on top of the reaction solution was used to avoid fibril sedimentation onto the array. This led to the identification of only one target significantly above the noise, namely glycogen synthase kinase 3α (Fig. 6b, c), as validated using thermophoresis, surface plasmon resonance and phosphorylation assays (Dunning et al. 2016).

## Monomer-dependent secondary nucleation of other Aβ variants

Amyloid β peptide exists in body fluids, such as blood and cerebrospinal fluid, as a range of length variants. The main species have 40 or 42 residues (Fig. 2), but there are numerous variants with fewer or more residues at the N- and C-termini (Kaneko et al. 2014; Welzel et al. 2014). A number of recent studies have shown that monomer-dependent secondary nucleation is a process pertinent not only to Aβ42 but also to Aβ40 (Meisl et al. 2014), N-terminally extended Aβ42 (Szczepankiewicz et al. 2015) as well as Aβ42 with familial mutations (Bolognesi et al. 2014; Meisl et al. 2016a; Yang et al. unpublished data). The rate of secondary nucleation can be enhanced upon reduced electrostatic repulsion between monomers and fibrils upon pH modulation (Meisl et al. 2016a) or salt screening (Abelein et al. 2016; Meisl et al. 2017). The relative importance of secondary nucleation may increase upon dominating suppression of other microscopic processes, such as elongation (Abelein et al. 2015) or primary nucleation (Meisl et al. 2014).

## Saturation of secondary nucleation reveals the multi-step nature of the process

**Fig. 7:**
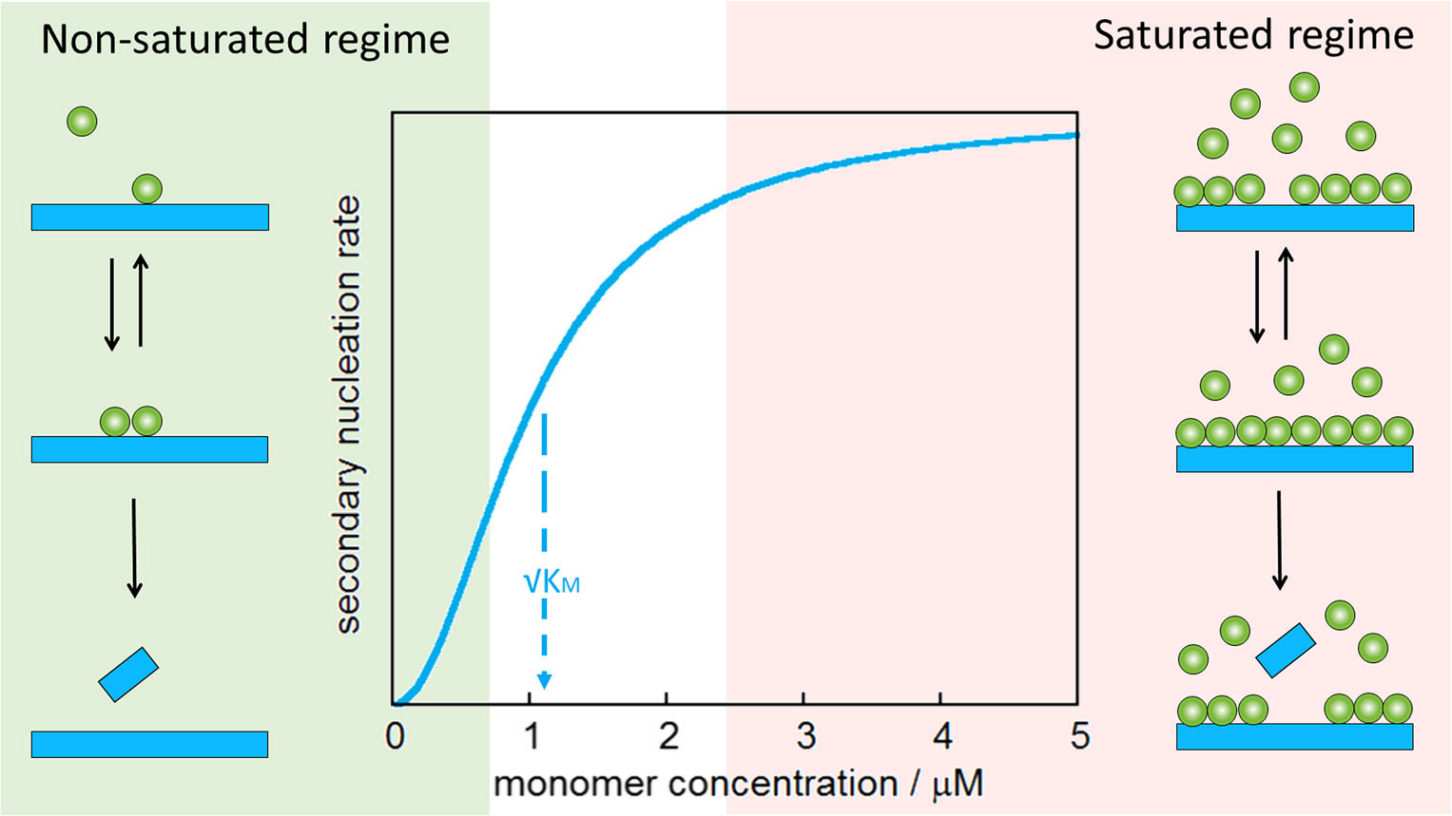
Multi-step secondary nucleation. The process of secondary nucleation can be decomposed into at least two steps: reversible binding of monomers at the fibril surface and product (fibril) formation (Meisl et al. 2014). This is similar to Michaelis–Menten kinetics of enzyme kinetics, displaying an unsaturated regime of strong rate dependence on monomer concentration at low monomer concentration (*left*) and a saturated regime at high monomer concentration where the rate of secondary nucleation becomes independent of monomer concentration (*right*). At a monomer concentration equal to √K_M_, the process is half saturated

In several cases, secondary nucleation is observed to saturate at high monomer concentration. Aβ40 was the first case of Aβ for which saturation of secondary nucleation was observed (Meisl et al. 2014), and this phenomenon has since also been observed for Aβ42 upon a change in pH (Meisl et al. 2016a, b), in human cerebrospinal fluid (Frankel et al., unpublished data), for some disease-associated mutants (Bolognesi et al. 2014; Meisl et al. 2016a; Yang et al. unpublished data) as well as for designed mutants (Sanagavarapu et al., unpublished data; Thacker et al., unpublished data). The observation of saturation of the rate of secondary nucleation at high monomer concentration reveals the multi-step nature of this process and Michaelis–Menten-like kinetics (Fig. 7). The composite steps may include association of monomer with aggregates, nucleation on the surface and detachment (Fig. 7). Depending on the ratio between monomer concentration and available surface area, any one of these steps may become rate-limiting at high monomer concentration. The kinetic modelling of saturated secondary nucleation includes the equivalent of a Michaelis constant, the square root of which indicates the monomer concentration at which the process is half saturated (Meisl et al. 2014) (Fig. 7). At low monomer concentration, the process is unsaturated (Fig. 7, left) and the observed overall aggregation profiles are strongly dependent on monomer concentration (as seen in Fig. 1a, for example). At high monomer concentration, the process is saturated (Fig. 7, right) and the observed overall aggregation profiles show little dependence on monomer concentration.

## Specificity

The 40- and 42-residue amyloid β peptide (Aβ40, Aβ42) seem to cross-react at the stage of primary nucleation, leading to acceleration of Aβ40 aggregation in the presence of Aβ42 monomers and a slight retardation of Aβ42 aggregation in the presence of Aβ40 monomers (Cukalevski et al. 2015 and references therein). Intriguingly, however, Aβ40 monomers fail to nucleate on fibrils of Aβ42 and Aβ42 monomers fail to nucleate on fibrils of Aβ40 (Cukalevski et al. 2015). This is a remarkable result which implies that monomer-dependent nucleation on fibrils is not a general surface effect but dependent on the detailed structure of the catalytic surface. On the contrary, N-terminally extended Aβ42 peptides cross-seed with normal Aβ42, implying that decoration of fibrils with extended N-termini does not interfere with the catalytic reaction, which suggests that the failure of Aβ40–Aβ42 cross-seeding may be due to differences in the packing of the C-terminus and thereby fibril core (Colvin et al. 2016).

## Inhibitors of secondary nucleation

**Fig. 8:**
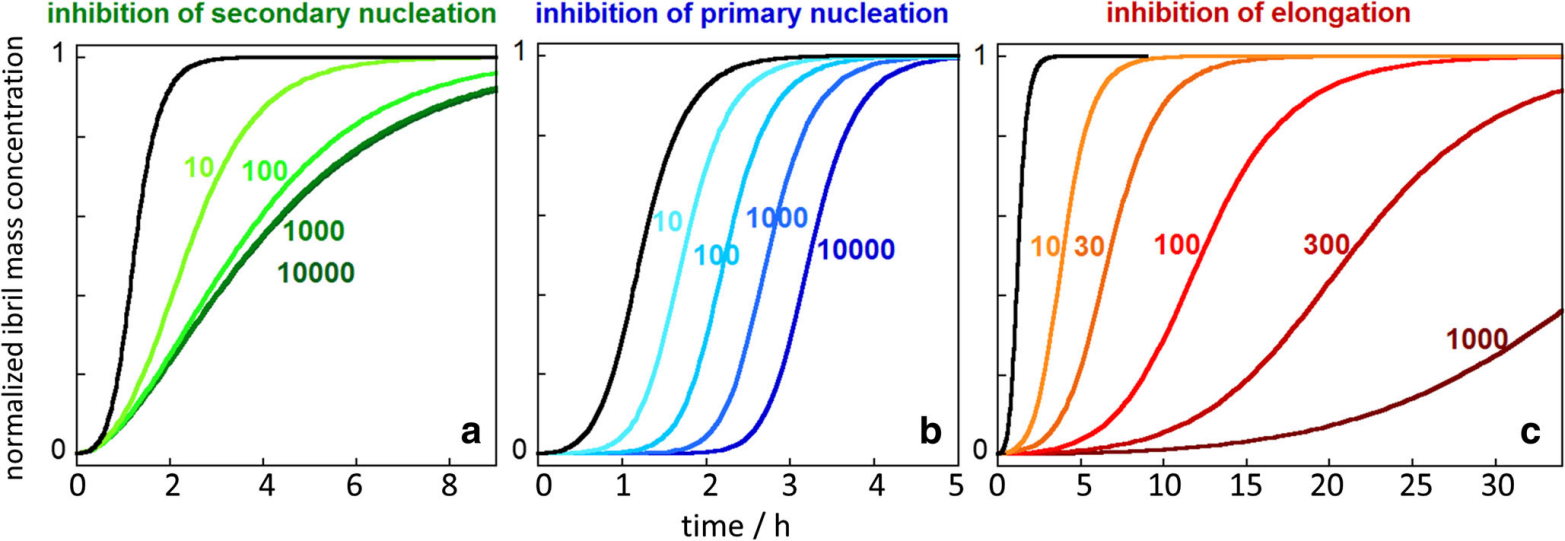
Changes in the macroscopic aggregation curves (fibril mass concentration as a function of time) upon selective reduction of the rate constants of: **a** secondary nucleation (k_2_), **b** primary nucleation (k_n_), **c** elongation (k_+_) calculated using the Amylofit platform (Meisl et al. 2016b). The reference *black curves* in each panel were calculated using the rate constants measured for Aβ42: k_n_ = 3^.^10^−1^ M^−1^ s^−1^, k_2_ = 1^.^10^4^ M^−1^ s^−1^, k_+_ = 3^.^10^6^ M^−1^ s^−1^ in 20 mM sodium phosphate buffer with 0.2 mM EDTA, pH 8.0, 37 °C (Cohen et al. 2013). For the calculation of each *coloured curve*, two rate constants were fixed at the values above and the *numbers* indicate fold-reduction of the selected microscopic rate constant (e.g. 10 indicates that the rate constant is reduced 10-fold). All calculations assumed that the reaction was initiated at time zero from a solution containing 3 μM Aβ42 monomer

Inhibitors may suppress a single microscopic step in the overall aggregation process, or they may act on more than one step, depending on whether the inhibitor interacts with monomers, oligomer or fibrils (Arosio et al. 2016). Fibril-binding molecules may block secondary nucleation or elongation. The screening for inhibitors that specifically suppress secondary nucleation is facilitated by the fact that very different effects on the macroscopic aggregation curves can be expected upon inhibition of this process (Fig. 8a) compared to inhibition of primary nucleation (Fig. 8b) or elongation (Fig. 8c). Inhibition of secondary nucleation leads to a reduced slope of the growth phase, and this effect saturates at high inhibitor concentrations (Figure 8A).

The first example of a specific inhibitor of monomer-dependent secondary nucleation is the molecular chaperone domain pro-SPC Brichos, which inhibits this process in a highly selective manner by binding to the fibril surfaces (Cohen et al. 2015). Electrophysiology measurements show that the toxicity related to oligomers generated in an ongoing aggregation reaction is blocked in the presence of pro-SPC Brichos (Cohen et al. 2015). Another example of secondary nucleation inhibitors is provided by a set of antibody fragments (scFvs) selected from phage-display libraries as fibril-specific binders using negative selection to remove monomer binders and positive selection to retain fibril binders (Munke et al. 2017). The coupling of phage display and kinetic screening allowed identification of antibody fragments that inhibit the secondary nucleation of Aβ42 on fibril surfaces in a specific manner, while disregarding scFvs that inhibit elongation to ensure suppression rather than enhancement of oligomer generation (Munke et al. 2017). Inhibition of secondary nucleation has also been observed with small molecules (Habchi et al. 2017) and designed antibodies (Aprile et al. 2017).

## Discovery of monomer-dependent secondary nucleation of α-synn

α-Synuclein, which is involved in Parkinson’s disease, displays monomer-dependent secondary nucleation at mildly acidic pH (Gaspar et al. 2017). For this protein, primary nucleation in bulk solution is extremely slow. To be able to observe aggregation within a reasonable experimental time-frame under quiescent conditions, one may use foreign surfaces, such as polystyrene surfaces in the form of nanoparticles (Vácha et al. 2014) or plates (Grey et al. 2011), or negatively charged phospholipid membranes (Galvagnion et al. 2015) to promote heterogeneous primary nucleation. Alternatively, aggregation can be monitored for solutions of monomer supplemented with a low concentration of premade fibrils to bypass the need for primary nucleation altogether (Buell et al. 2014; Gaspar et al. 2017). In the case of α-syn, the mechanism of fibril propagation and growth is dependent on pH. At neutral pH, the reaction is dominated by elongation of seeds, whereas at pH below 6.0, a secondary process is significant (Buell et al. 2014). The existence of secondary nucleation was thus first inferred from the requirement of a secondary process to produce reasonable global fits to aggregation data over a range of protein concentrations and the significant shortening of the lag phase upon addition of low (sub-%) concentrations of seeds (Buell et al. 2014). However, as described above for Aβ42, additional experiments are needed to assess whether this secondary process can be ascribed to the nucleation of monomers on the surface of fibrils. Gaspar et al. (2017) used a combination of trap-and-seed experiments, quartz crystal balance with dissipation and centrifugal sedimentation analyses of size distribution, and the data revealed that the secondary process observed at mildly acidic pH is indeed monomer-dependent secondary nucleation on the surface of fibrils.

## Questions for the future

Although, several aspects of monomer-dependent secondary nucleation have been revealed, large number of intriguing questions regarding secondary nucleation in amyloid formation remain to be addressed in future work. These include the following:

- *General or specific phenomenon?* Is surface-catalysed secondary nucleation a general phenomenon pertinent to most or all self-assembly reactions of amyloid peptides or is it a specific phenomenon present in certain systems only?
- *Nature of the catalytic “sites”—specific or diffuse?* Does secondary nucleation occur at, or require, specific catalytic sites on the fibril surface, or is this a more diffuse surface phenomenon dependent on the overall molecular character of the surface?
- *Molecular determinants of secondary nucleation*. Can any general rules be deciphered about the sequence requirements for secondary nucleation? Which molecular features of the surface increase or decrease the rate of secondary nucleation? Are specific amino acid side chains or back-bone features involved? Are there any general rules regarding the level of hydrophobicity, hydrophilicity or charge of the fibril surface and monomer, respectively? What is the role of the environment in terms of pH, temperature, pressure and ionic strength?
- *Relation between surface affinity and secondary nucleation*. Is there an optimal affinity between monomer and surface, between nucleus and surface or between oligomers and surface, for the reaction to be productive? Is the process under thermodynamic or kinetic control? Which fraction of nucleated species does eventually form fibrils?
- *Size and structure of newly nucleated and detaching species?* What is the structure of nucleated species? How large do oligomers grow before they detach? Does the conversion from oligomer to fibril happen on the fibril surface or in solution after detachment? What is the minimal set of composite steps needed to describe the secondary nucleation of monomers on fibrils surface? At what stage in the process do two or more filaments come together in a wind-around-each-other fibrillar type assembly? Do multiple filaments grow in parallel?
- *Propagation of aggregate morphology—origin of the strain phenomenon?* Does secondary nucleation lead to the proliferation of amyloid aggregates of a defined morphology, i.e. is the so-called strain phenomenon rooted in secondary nucleation? It is well-known that seeding of a supersaturated monomer solution with pre-formed crystals leads to the generation of new crystals having the same morphology, chirality, crystal packing and space group as the seed (Botsaris 1976; Kondepudi et al. 2010). In the prion and amyloid field, this is often discussed as being due to fragmentation and growth of the broken seeds; this view may at least in part have originated from the fact that many experiments have been performed under vigorous mechanical agitation, conditions under which the generation of new aggregates is in fact dominated by fragmentation (Knowles et al. 2009). A key question is whether the strain phenomenon in amyloid formation and prion propagation (Orgel 1996) might also, at least in some systems, be a consequence of secondary nucleation.
- *Relevance in vivo?* Finally, the importance of secondary nucleation in vivo remains to be found. This is a highly relevant and intriguing question. Primary nucleation in vivo is most likely dominated by heterogeneous primary nucleation at a multitude of surfaces; the rate of this process will not change after emergence of the first aggregates of the particular protein as long as the monomer concentration remains unchanged. The autocatalytic nature of secondary nucleation, however, may make this process more and more critical the more aggregates that have already emerged. The relative importance of secondary versus primary nucleation in vivo is still very difficult to predict as each step is attenuated by the presence of multiple chaperones, and the power of such attenuation will vary over time. Chaperones and other mechanisms act in concert to prevent the organism by collectively suppressing all steps in the aggregation process, although several of the chaperones on their own seem to inhibit a distinct microscopic step (Arosio et al. 2016 and references therein).
